# 1324. Identification of Local Risk Factors for *P. aeruginosa* in Community-acquired Pneumonia in a Veteran Population

**DOI:** 10.1093/ofid/ofab466.1516

**Published:** 2021-12-04

**Authors:** Emily A Gibbons, Teri L Hopkins, Linda Yang, Christopher R Frei, Marcos I Restrepo, Elizabeth Walter, Jose Cadena-Zuluaga

**Affiliations:** 1 South Texas Veterans Health Care System, San Antonio, Texas; 2 South Texas Veterans Health Care System, UT Health San Antonio, UT Austin College of Pharmacy, Baltimore, Maryland; 3 University of Texas at Austin College of Pharmacy/UT Health San Antonio, San Antonio, Texas; 4 University of Texas health and science center San Antonio, Audie L. Murphy VA Medical Center, San Antonio, Texas

## Abstract

**Background:**

The 2019 ATS/IDSA community-acquired pneumonia (CAP) guidelines recommend empiric *P. aeruginosa* (PSA) coverage if locally validated risk factors are present. They further recommend obtaining local data on CAP pathogens to quantify risk factors and help guide clinical decision-making. To comply with the current guideline recommendations and to determine which patients may benefit from empiric anti-pseudomonal therapy, we aimed to characterize our institution’s local risk factors for CAP caused by PSA.

**Methods:**

This is a retrospective single-center matched cohort study of patients admitted to our institution with a CAP diagnosis and a positive respiratory culture who received antibiotic treatment in the past 19 years. Multivariate logistic regression was performed to assess the relationship between PSA and the following risk factors: severe or very severe COPD (FEV1 < 50% predicted), requiring invasive mechanical ventilation or vasopressor support in the first 24 hours of admission, history of PSA infection/colonization in the previous year, tracheostomy, bronchiectasis, long-term care facility residence and admission with receipt of IV antibiotics in the previous 90 days.

**Results:**

A total of 343 patients were screened and 213 were included. Patients were mostly male (99%) with a median (IQR) age of 70 (63-76) years. Long-term care facility residence was removed from the model to prevent it from being over fit as it was related tracheostomy. In the multivariate analysis the only independently associated risk factor for PSA CAP was evidence during the prior year of PSA infection or colonization (OR 3.66; 95% CI 1.26 – 10.56; p = 0.018). Other risk factors that did not reach statistical significance but may be clinically significant included severe or very severe COPD (OR 2.52; 95% CI 2.52 – 6.38; p = 0.055) and tracheostomy (OR 5.28; 95% CI 0.74 – 38.85; p = 0.098).

**Conclusion:**

The results of this study provide valuable data to help guide empiric CAP treatment at our institution. Based on these results, patients with PSA infection or colonization in the past year are appropriate to provide empiric anti-pseudomonal therapy for CAP. Further evaluation of severe or very severe COPD and tracheostomy would be beneficial to better characterize their role in PSA CAP.

**Disclosures:**

**All Authors**: No reported disclosures

